# Supplementary material for: A Critical View of the Application of the APEX Software (Aqueous Photochemistry of Environmentally-Occurring Xenobiotics) to Predict Photoreaction Kinetics in Surface Freshwaters
Source: Molecules. 2019 Dec 18;25(1):9. doi: 10.3390/molecules25010009 (PMC7017383; doi:10.3390/molecules25010009)
Supplement: Supplementary file 1 [file molecules-25-00009-s001.zip › 3.2.4_gcc-4.4.0.zip!/3.2.4_gcc-4.4.0/3.2.4_gcc-4.4.0/doc/octave/html/interpreter/The-_003ccode_003efor_003c_002fcode_003e-Statement.html]

The @code{for} Statement - Untitled


Next: The <code>break</code> Statement,
Previous: The <code>do-until</code> Statement,
Up: Statements


---

### 10.5 The `for` Statement

The `for` statement makes it more convenient to count iterations of a
loop. The general form of the `for` statement looks like this:

```
     for var = expression
       body
     endfor
```

where body stands for any statement or list of statements,
expression is any valid expression, and var may take several
forms. Usually it is a simple variable name or an indexed variable. If
the value of expression is a structure, var may also be a
vector with two elements. See Looping Over Structure Elements, below.

The assignment expression in the `for` statement works a bit
differently than Octave's normal assignment statement. Instead of
assigning the complete result of the expression, it assigns each column
of the expression to var in turn. If expression is a range,
a row vector, or a scalar, the value of var will be a scalar each
time the loop body is executed. If var is a column vector or a
matrix, var will be a column vector each time the loop body is
executed.

The following example shows another way to create a vector containing
the first ten elements of the Fibonacci sequence, this time using the
`for` statement:

```
     fib = ones (1, 10);
     for i = 3:10
       fib (i) = fib (i-1) + fib (i-2);
     endfor
```

This code works by first evaluating the expression `3:10`, to
produce a range of values from 3 to 10 inclusive. Then the variable
`i` is assigned the first element of the range and the body of the
loop is executed once. When the end of the loop body is reached, the
next value in the range is assigned to the variable `i`, and the
loop body is executed again. This process continues until there are no
more elements to assign.

Within Octave is it also possible to iterate over matrices or cell arrays
using the `for` statement. For example consider

```
     disp("Loop over a matrix")
     for i = [1,3;2,4]
       i
     endfor
     disp("Loop over a cell array")
     for i = {1,"two";"three",4}
       i
     endfor
```

In this case the variable `i` takes on the value of the columns of
the matrix or cell matrix. So the first loop iterates twice, producing
two column vectors `[1;2]`, followed by `[3;4]`, and likewise
for the loop over the cell array. This can be extended to loops over
multidimensional arrays. For example

```
     a = [1,3;2,4]; b = cat(3, a, 2*a);
     for i = c
       i
     endfor
```

In the above case, the multidimensional matrix c is reshaped to a
two-dimensional matrix as `reshape (c, rows(c),
prod(size(c)(2:end)))` and then the same behavior as a loop over a two
dimensional matrix is produced.

Although it is possible to rewrite all `for` loops as `while`
loops, the Octave language has both statements because often a
`for` loop is both less work to type and more natural to think of.
Counting the number of iterations is very common in loops and it can be
easier to think of this counting as part of looping rather than as
something to do inside the loop.

- Looping Over Structure Elements
